# Pre-Existing Mutations in Reverse Transcriptase of Hepatitis B Virus in Treatment-Naive Chinese Patients with Chronic Hepatitis B

**DOI:** 10.1371/journal.pone.0117429

**Published:** 2015-03-30

**Authors:** Jie Xu, Biao Wu, Jing-Hui Wang, Ling Huang, Deng-yu Wang, Ling Zhao, Guo-ping Zhao, Ying Wang

**Affiliations:** 1 Department of Infectious Diseases, No.3 People’s Hospital affiliated to Shanghai Jiao Tong University School of Medicine, Shanghai, China; 2 Shanghai Institute of Immunology, Shanghai Jiao Tong University School of Medicine, Shanghai, China; 3 Shanghai Key Laboratory of Health and Disease Genomics, Chinese National Human Genome Center at Shanghai/Shanghai Academy of Science and Technology, Shanghai, China; University of Athens, Medical School, GREECE

## Abstract

High rate of viral replication and lacking of proofreading activity in hepatitis B virus (HBV) polymerase lead to the generation of mutations in HBV virus. Mutations in the reverse transcriptase (RT) region of HBV polymerase are demonstrated to be strongly associated with drug resistance during antiviral treatment. However, the presence of mutations as well as its clinical significance in treatment-naïve hepatitis patients (defined as pre-existing mutations) need to be further investigated. In the present study, a total of 168 serum samples from treatment-naive chronic hepatitis B (CHB) patients were collected, and the RT region of HBV polymerase was sequenced. The results showed that pre-existing mutations in the RT region of HBV polymerase were detected in 43 of 168 (25.6%) treatment-naive CHB patients within which there were no well-characterized primary nucleotide analogs (NAs) resistance sites. Three dominant sites at rt191, rt207 and rt226 were found mutant in 7(16.28%), 8(18.60%), and 14(32.56%) samples respectively among these 43 patients. No significant correlation was found between pre-existing mutations and gender, age, HBV genotype, ALT, HBeAg or HBV DNA loads. However, patients with pre-existing RT mutations under HBeAg sero-negative status exhibited decreased HBV DNA loads, which contributed to the decreased HBV DNA loads in the total HBeAg sero-negative patients. The above investigation indicated that there was a prevalence of pre-existing mutations in RT region of HBV polymerase which might affect the serum HBV DNA level in treatment-naive CHB patients. Its effects on the occurrence of NAs resistance and the prognosis after treatment need to be further investigated.

## Introduction

Chronic hepatitis B virus (HBV) infection, a major cause of cirrhosis and hepatocellualr carcinoma, afflicts approximately 350 million people worldwide[1] and 93 millions of Chinese population[2]. HBV is a partially double-stranded DNA virus containing 3200 nucleotides with four open reading frames (ORFs), encoding pre-S/S, pre-C/C, HBX and polymerase. It replicates DNA genome through RNA intermediate during viral life cycle using reverse transcriptase encoded by DNA-P region[3]. Lacking of proof reading ability of reverse transcriptase, the error rate after the replication of viral genome has thus been mounted as 10^−7^ per nucleotide which is 10-fold higher than other DNA virus[4]. Mutations accumulated in the individual genome reflect the selection pressure either from virus or host immune responses. In addition, antiviral treatment represents exogenous selection to determine the survival advantage of HBV mutants[5]. High rate of mutations in HBV genome compromises the antiviral therapy of nucleotide analogs (NA), leading to the generation of drug resistant viral strains and disease progression.

HBV polymerase contains four domains as terminal protein, spacer, reverse transcriptase and ribonuclease H. According to the latest consensus of nomenclature system[6], reverse transcriptase of HBV polymerase consists of 344 amino acids starting with the highly conserved EDWGPCDEHG motif and partially overlaps with HBV surface antigens. Mutations within reverse transcriptase probably affect the replication capacity of HBV, which in turn might alter the antigenicity, encapsidation and virulence of virus as well as the generation of drug resistance[7–9]. Indeed, the drug resistant mutations in reverse transcriptase were extensively explored during antiviral therapy of NAs, including lamivudine(LMV)[10], adefovir (ADV) [11], entecavir (ETV) [12] and telbivudine (LdT) [13]. It is reported that LMV-resistant strains were detected in about 70% of patients after 5 years of treatment with mutant sites at rtM204I/V [14]. Mutant strains with ADV resistance anchor at rtA181T/V and rtN236T [15]. The virological significance of some mutants is also investigated [16, 17].

However, the genetic variability in treatment-naïve chronic hepatitis B patients was unthoroughly investigated. Defining the mutations in treatment naïve patients as pre-existing mutations, the clinical characteristics and significance of these pre-existing mutations were investigated in the present study.

## Results

### Clinical features of treatment-naïve CHB patients

Samples from 168 treatment-naive CHB patients were enrolled in this retrospective study. Their clinical features were summarized in Table 1. The patient cohort consisted of 77.38% males and 22.62% females with a median age of 40 years (range 19–81 years). High serum ALT level (≥10N) was found in 33.73% patients. 61.31% of the subjects displayed high HBV DNA level in the serum (≥10^6^ copies/mL) while 64.88% of them were HBeAg sero-positive.

**Table 1:** Clinical features of the 168 treatment-naive CHB patients.

Characteristic	Number (Percentage)
Gender (male/female)	130/38 (77.38%/22.62%)
years of Age, median (range)	40 (19–81)
Genotype (type B/C)	68/100 (40.48%/59.62%)
ALT* (≥10N/<10N)	56/110 (33.73%/66.27%)
AST* (≥10N/<10N)	33/133(24.81%/75.19%)
TBIL* (μmol/L), median (range)	33(6.2–612.6)
HBV DNA (≥10^6^/<10^6^ copies/mL)	103/65 (61.31%/38.69%)
Serum HBeAg (positive/negative)	109/59 (64.88%/35.12%)

ALT, alanine aminotransferase; HBeAg, hepatitis B e antigen;

*N = 166.

### Correlation between pre-existing mutations and clinical features

Through gene sequencing and sequence alignment with 10 standard genotype (A-J) strains in Genbank, HBV genotypes of the subjects were determined with either type B or C in which type C was the majority (59.62%) (Table 1). Among 168 samples tested, 43 samples (25.6%) had pre-existing mutations in the HBV RT region. The correlation between pre-existing mutations and clinical features, including HBV genotype, age, gender, HBeAg, ALT level and baseline serum HBV DNA in 168 treatment-naïve CHB patients were further analyzed. As indicated in Table 2, no significant correlations were found between the incidence of pre-existing mutations and those clinical features (all P > 0.05, Table 2).

**Table 2:** Epidemiological analysis of pre-existing mutations in HBV RT region.

	Group with mutations	Group without mutations	*P*-value
	(*n* = 43)	(*n* = 125)	
Genotype Type B	17	51	0.884
Type C	26	74	
Gender Male	36	94	0.249
Female	7	31	
HBeAg Positive	27	82	0.739
Negative	16	43	
Age ≥40 years old	25	63	0.381
<40 years old	18	62	
HBV DNA ≥10^6^ copies/mL	22	81	0.113
<10^6^ copies/mL	21	44	
ALT * ≥10N	12	44	0.348
<10N	31	79	

HBeAg, hepatitis B e antigen; HBV, hepatitis B virus; ALT, alanine aminotransferase;

*N = 166.

### Characterization of pre-existing mutations in RT regions from treatment-naïve CHB patients

**Fig 1:**
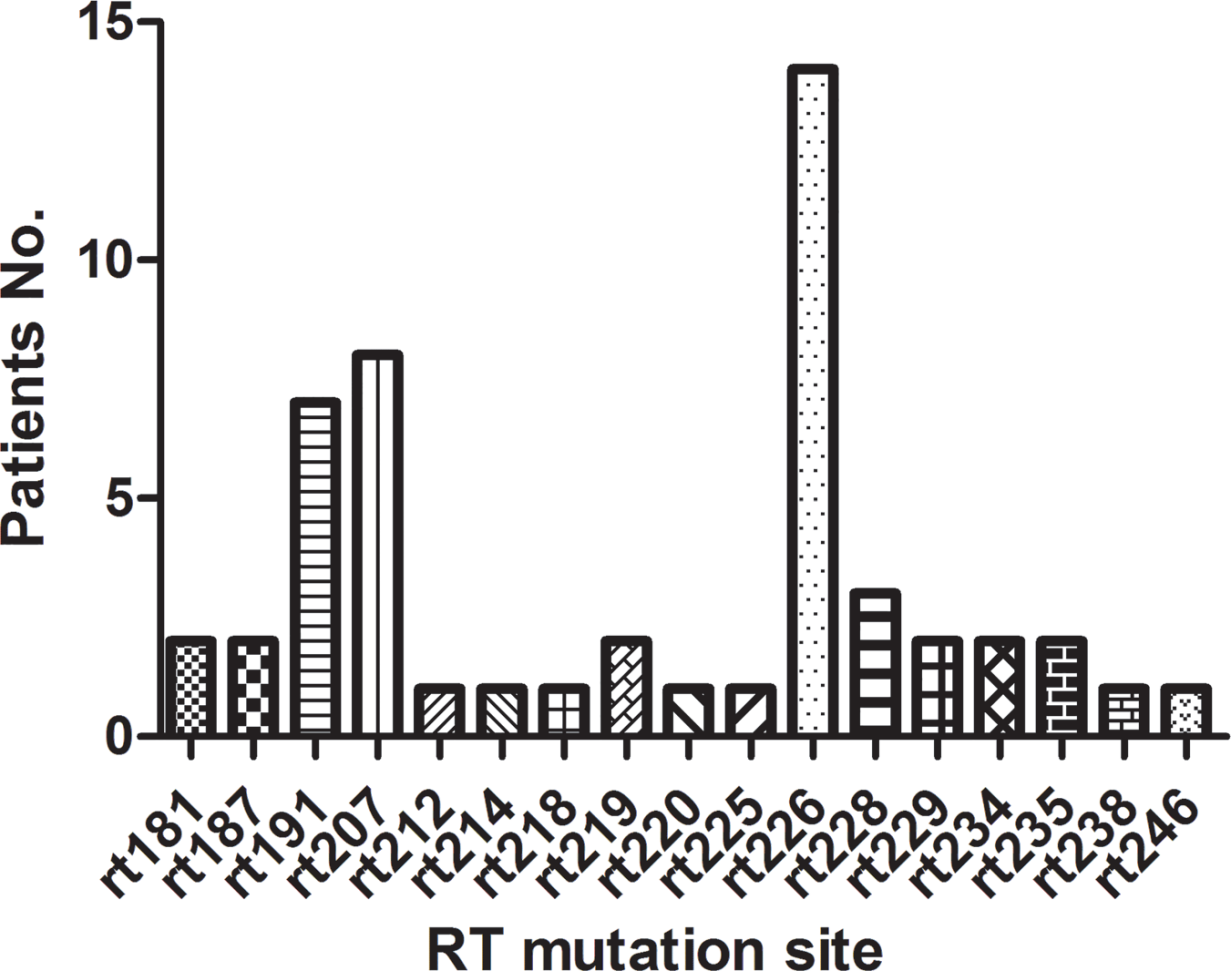
Distribution of pre-existing mutations in HBV RT region of 43 samples.

All of the amino acid substitutions detectable in 43 samples with pre-existing mutations in RT region are listed in Table 3. No well-characterized primary nucleotide analogs (NAs) resistant mutations (i.e. I169T, A181T/V, T184A/C/F/G/I/L/M/S, A194T, S202C/G/I, M204I/V/S, N236T, M250I/L/V) or secondary/compensatory mutations (i.e. L80I/V, V173L, L180M) [19, 20] were found in these mutations. Within 43 samples, 35 of them (81.40%) had single amino acid substitution, 7 patients (16.28%) had two amino acids substitution, and 1 patient (2.33%) had three amino acid substitutions. There were a total of 17 pre-existing mutant sites detectable among 43 samples, in which three dominant mutation sites rt191, rt207 and rt226 were present in 7(16.28%), 8(18.60%), and 14(32.56%) samples respectively (Fig 1).

**Table 3:** Characteristics of amino acid mutations in HBV RT region.

Characteristics	Genotype	No.
rtA181S	C	2
rtI187V	B	1
rtI187V + rtN226H	B	1
rtV191I	C	7
rtL207V	B	1
rtL207V + rtN226H	B	1
rtV207M	C	1
rtV207M + rtV214A	C	1
rtV207M + rtN226H	C	3
rtV207I + rtN226H	C	1
rtK212R	B	1
rtV214A	C	1
rtE218D	B	1
rtS219A	C	1
rtS219A + rtT225V + rtN226H	B	1
rtL220I	B	1
rtN226H	B	6
rtN226T	C	1
rtL228F	B	2
rtL228I	C	1
rtL229V	C	2
rtH234R	B	1
rtH234Q	C	1
rtL235I	C	2
rtN238G	C	1
rtS246C	B	1

RT, reverse transcriptase.

### Patients with pre-existing Mutations in RT region showed decreased HBV DNA levels within HBeAg sero-negative group

**Fig 2:**
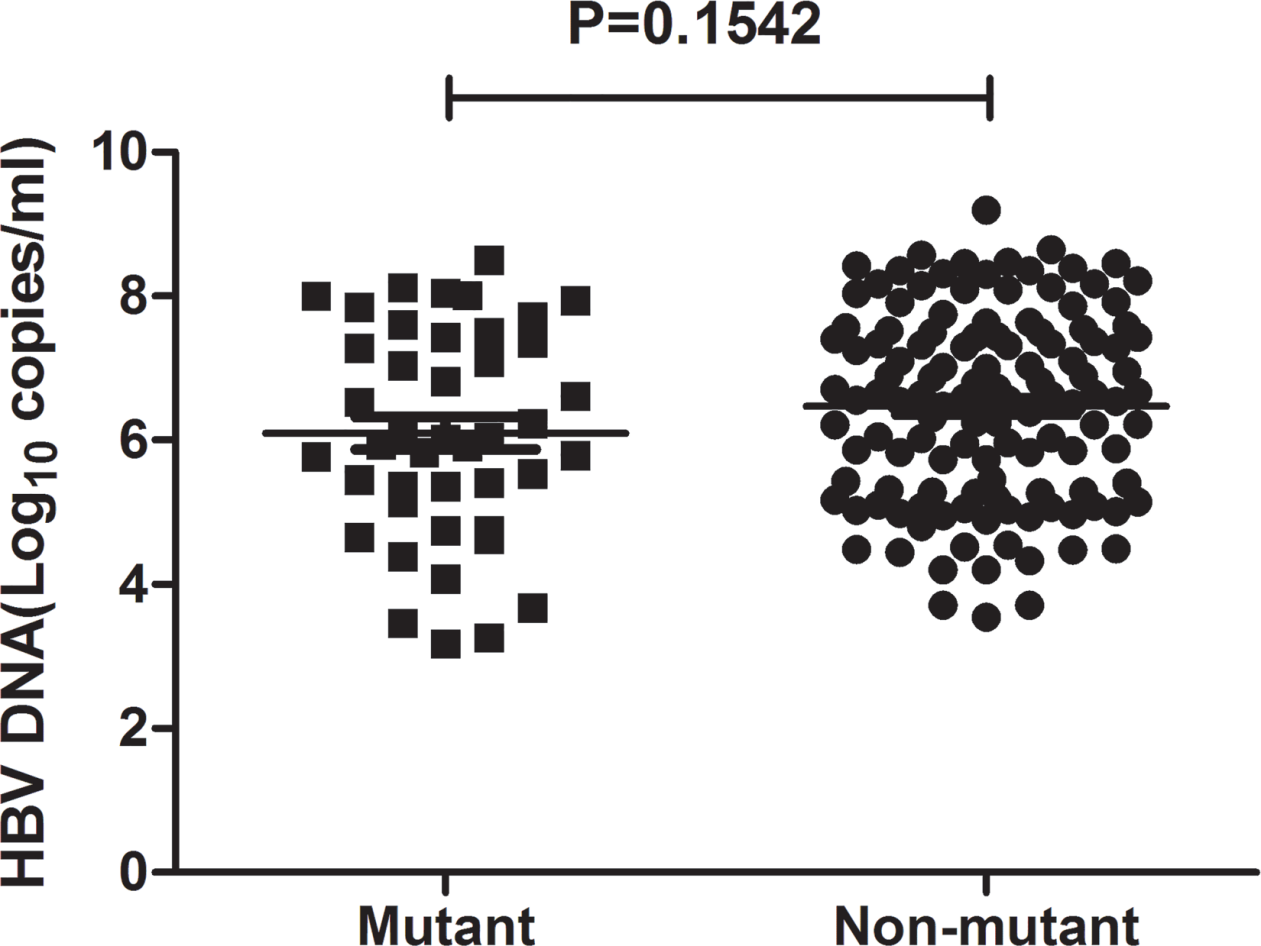
HBV DNA levels in 168 treatment-naïve CHB patients with or without pre-existing RT mutations.

**Fig 3:**
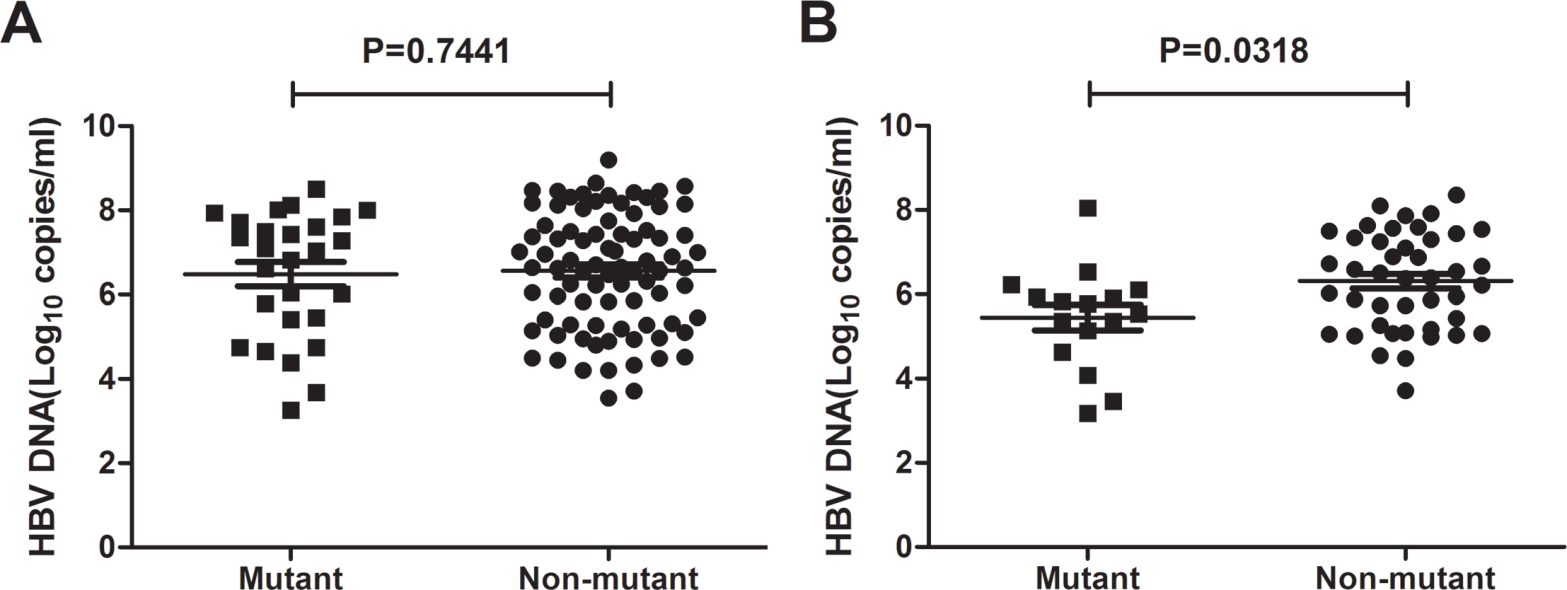
HBV DNA levels in CHB patients with or without pre-existing RT mutations among HBeAg sero-positive group (A) and HBeAg sero-negative group(B).

In this study, pre-existing RT mutations were associated with decreased serum HBV DNA level within the total treatment-naïve patients, but the difference didn’t reach significance (mutant *vs*. non-mutant: 10^6.08^ *vs*. 10^6.47^, *P* = 0.113, Fig 2). However, when we sub-grouped the patients into HBeAg sero-positive and HBeAg sero-negative groups, it turned out that the average HBV DNA loads were significantly lower in patients with pre-existing RT mutations when comparing with non-mutant ones within HBeAg sero-negative group (mutant *vs*. non-mutant: 10^5.45^ *vs*. 10^6.23^, P = 0.0318, Fig 3B). The serum HBV DNA loads were comparable between mutant and non-mutant patients within HBeAg sero-positive group (mutant *vs*. non-mutant: 10^6.46^ *vs*. 10^6.60^, P>0.05, Fig 3A).

### Pre-existing RT Mutations contribute to the decreased viral loads in treatment-naïve HBeAg sero-negative patients

**Fig 4:**
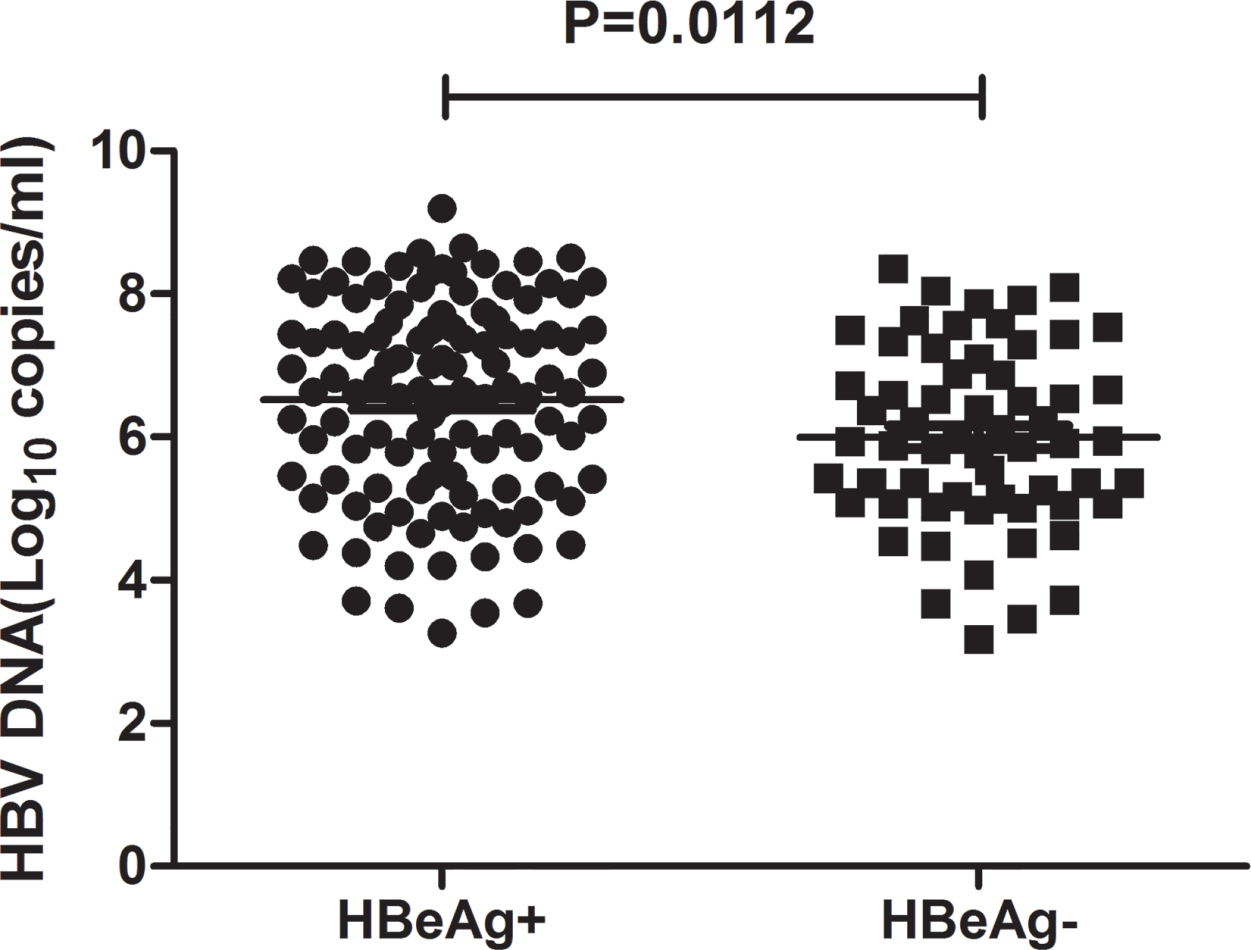
HBeAg sero-negative patients displayed lower HBV DNA level than HBeAg sero-positive patients.

**Fig 5:**
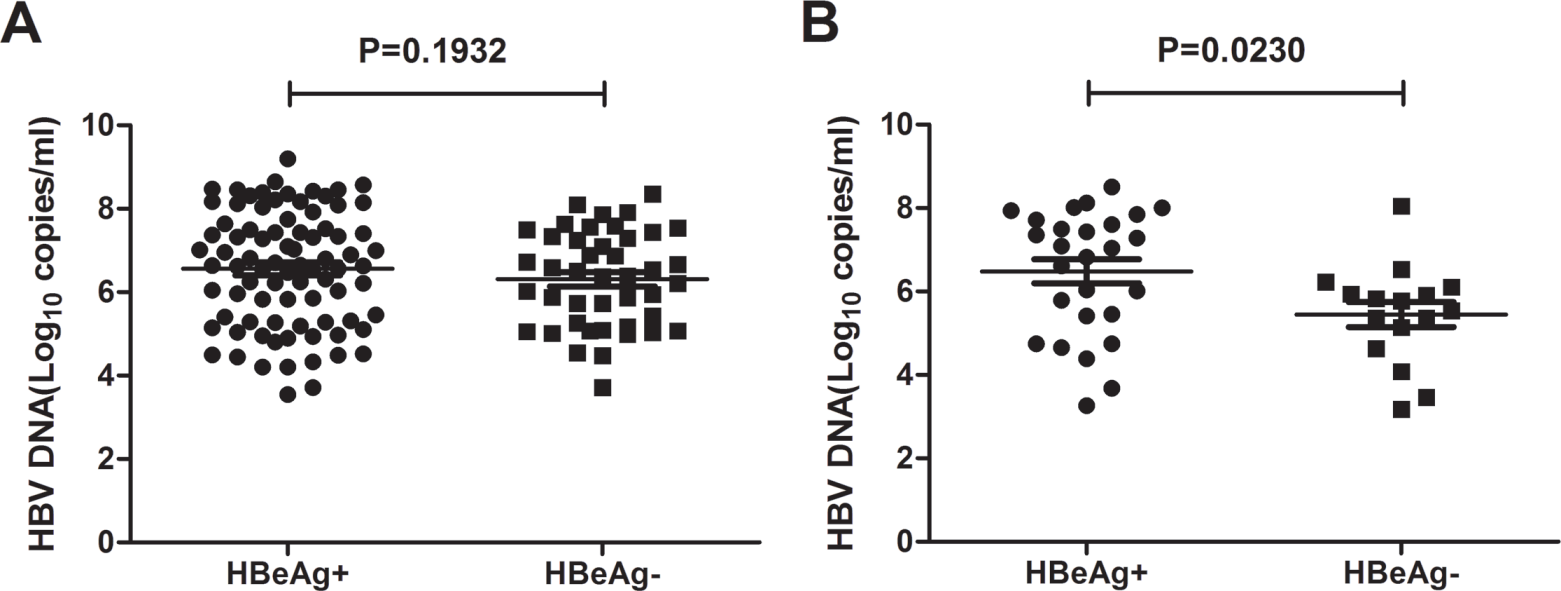
HBV DNA levels in HBeAg sero-positive and HBeAg sero-negative groups among patients without pre-existing RT mutations (A), and patients with pre-existing RT mutations (B).

It is known that there is a direct relationship between HBeAg positivity status and high levels of HBV DNA loads. But in HBeAg negative CHB patients, there are always precore/core mutations which could restore or increase the viral loads. Within the total 168 samples in this study, HBeAg sero-negative patients showed decreased viral loads (HBeAg^+^ vs HBeAg^-^: 10^6.565^ vs 10^6.020^, P = 0.0112, Fig 4). On the other hand, when excluding the 43 patients with pre-existing RT mutations and comparing HBV DNA loads in the 125 patients without mutations, no significant difference were found between HBeAg sero-positive group and HBeAg sero-negative group(HBeAg^+^ vs HBeAg^-^: 10^6.646^ vs 10^6.252^, P = 0.1932, Fig 5A). Within the 43 patients with pre-existing RT mutations, HBeAg sero-negative group showed decreased viral loads (HBeAg^+^ vs HBeAg^-^: 10^6.463^ vs 10^5.445^, P = 0.023, Fig 5B). These results demonstrate that pre-existing RT mutations contribute to decreased HBV DNA loads in treatment-naïve HBeAg sero-negative patients.

## Discussion

Determination of mutant sites in HBV RT region is extensively applied to monitor drug resistance to NAs in the long-term treatment for CHB patients. There are certain well-known sites in HBV RT region as indicators for the resistance to lamivudine (rtV173L, rtL180M, rtM204V/I and rtA181V/T), adefovir (rtA181V/T and rtN236T) or entecavir (rtI169T, rtI184G, rtS202G/I, and rtM250V) etc, which provide important guidance to drug selection for clinicians[21, 22]. The existence of pre-existing mutations in RT region of HBV polymerase was also reported in previous literatures, for instance, secondary/compensatory but not primary mutations were found in treatment-naïve Italian population[23], while there were 8.8% Indian treatment-naïve CHB patients carrying primary drug resistant mutations[24]. Based on our retrospective study, we have demonstrated that pre-existing mutations in HBV RT gene exist in 25.6%(43/168) of treatment-naïve hepatitis B patients. Primary NAs resistance mutations or secondary/compensatory mutations were not found in this study, which is consistent with similar studies on Chinese population[19]. This might be due to the limitation of the sequencing approach which can not detect minor variants with a frequency below 20% or due to the property of the patient cohort enrolled in this study, because YMDD mutations were found in 26.9% Chinese CHB patients if the inclusion criteria were non-taking antiviral drugs within the last one year[25]. However, our result indicated that the naturally occurring NAs resistant strains were not prevalent or predominant in treatment-naïve Chinese CHB patients infected by B- or C-genotype virus.

Although primary or secondary/compensatory NAs resistance mutations were not found in this studied population, there were a lot of putative NAs resistant mutations which have been reported to be selected during prolonged NAs treatment or potentially associated with NAs resistance or compensatory replication capacity. For instance, rtV191I, rtV207I, rtL229V were associated with lamivudine resistance[26–28], and rtA181S, rtV214A, rtE218D were associated with adefovir resistance[29–31]. Besides NAs resistance, many mutations such as rtV191I, rtV207I, rtK212R, rtS219A, rtT225V, rtN226H caused accompanied mutations on the overlapped s gene and produced amino acids mutant or truncated HBsAg[32, 33]. This may support survival advantages by reducing antigenicity of the HBsAg protein under the natural selection pressure of hosts’ immune system in these treatment-naïve patients. What’s more, rtA181S, rtI187V, rtV191I and rtL229V were proved *in vitro* with decreased replication efficacy[7,28,34,35], while rtL220I, rtL228F/I, rtH234R and rtL235I were novel mutations of which biological or clinical significance has not been reported so far as we know.

Pre-existing mutations in HBV RT region are not apparently related to most of the clinical features, including HBV genotypes, age, gender, serum HBeAg, ALT level as well as baseline serum HBV DNA level. However, when we sub-grouped the patients into HBeAg sero-positive and sero-negative groups, the presence of pre-existing mutations in HBV RT region is dramatically related to the decrease of HBV DNA level within HBeAg sero-negative patients but not HBeAg sero-positive patients. This is quite unexpected. How HBeAg sero-negativity affects HBV DNA level in patients with pre-existing mutations remains unclear.

HBeAg is a non-structural protein encoded by gene HBV preC/C gene. It is derived from HBV core antigen (HBcAg) with the loss of some amino acid residues[36]. After synthesized in the cytoplasm, it is secreted out of liver cells through endoplasmic reticulum and distribute in the periphery blood and the whole body fluid[37, 38]. HBeAg is dispensable for the assembly and the replication of HBV, but it is demonstrated to be an indicator for rapid replication of HBV *in vivo* and high contagion. Serological conversion of HBeAg is a critical indicator for effectiveness of anti-viral drugs. After treatment, the decrease of virus DNA level accompanied by sero-conversion of HBeAg always indicates a good prognosis. Biologically, it is suggested that HBeAg induces immune tolerance when it is secreted into the serum[39]. HBeAg was reported to be able to induce the secretion of Th2 cytokines such as IL- 4, IL-10 in peripheral blood lymphocytes[40]. Peripheral circulation of HBeAg in transgenic mice could eliminate HBcAg- and HBeAg-specific Th1 cells through Fas-FasL mediated mechanism[41]. Whether the decline of HBV DNA level in HBeAg negative patients is owing to the recovery of Th1 cellular immune responses against HBeAg or related protein (such as HBcAg) still needs to be further investigated.

It has been reported that HBV RT mutations can affect HBV DNA replication. For instance, when the proline residue at position306 (rtP306) in HBV RT region is replaced by serine or other amino acid residues, virus replication efficacy declines[42]. It is also reported that the substitution of YMDD motif of ayw subtypes into YIDD, YVDD reduced the capacity of virus replication *in vitro*[43]. In addition, rtA194T mutation in HBV polymerase region results in a decreased HBV DNA replication capacity. However, when pre-C/BCP mutations co-exist with rtA194T mutation in HBeAg sero-negative status, the virus replication level will recover to the comparable levels of wild type strain[44]. But recent studies showed that pre-C/BCP mutations could only counteract the effects of rtA194T mutation. If other mutant sites exist in RT region meanwhile, the capacity of HBV DNA replication reduced as well[45]. In our study, we found that pre-existing mutations in HBV RT region was closely related to lower HBV DNA levels in HBeAg sero-negative subjects, which is consistent with impaired replication capacity of mutant stains such as rtA181S, rtI187V, rtV191I and rtL229V mutant stains[7,28,34,35].

In conclusion, we demonstrate that the presence of pre-existing mutations in RT region in treatment naïve CHB patients. These mutations might contribute to the decreased HBV DNA loads in HBeAg sero-negative patients. Its effects on the occurrence of NAs resistance and the prognosis after treatment need to be further investigated.

## Materials and Methods

### Clinical samples

This study was retrospective. A total of 168 treatment-naïve chronic HBV patients who visited Shanghai 3rd People’s Hospital Affiliated to Shanghai Jiao Tong University School of Medicine from July 2008 to May 2010 were enrolled in the study. All of the patients were first diagnosed as chronic hepatitis B and confirmed without taking any Nucleos(t)ide analogues. The diagnostic criteria were based on 2000 Xi’an Viral Hepatitis Management Scheme issued by the Chinese Society of Infectious Diseases and Parasitology, and the Chinese Society of Hepatology, Chinese Medical Association. The inclusion criteria of chronic hepatitis B patients included hepatitis B surface antigen (HBsAg) positive for more than 6 months and abnormal ALT level; while the exclusion criteria included hepatitis C virus or human immunodeficiency virus co-infection, autoimmune liver disease, and alcohol or drug abuse. Patients were informed and agreed to authorize the hospital to deal with their blood, tissue fluid and tissue sample for diagnosis and research with written consent. The study was approved by Ethics Committee of Shanghai 3^rd^ People’s Hospital. Patients’ serum was collected and frozen at -80°C.

### HBV serology and Liver biochemistry assays

The serology and liver biochemistry assays were routinely detected in the clinical laboratory of Shanghai 3^rd^ People’s Hospital affiliated to Shanghai Jiao Tong University School of Medicine. Serum hepatitis B s antigen (HBsAg), hepatitis B e antigen (HBeAg), anti-HBs, anti-HBe, anti-HBc were detected according to the instruction of the attached original commercial kits on the wholly automatic immune fluorescence analyzer Abbott Type I2000 (Abbott Laboratories, Illinois, USA). Serum alanine aminotransferase (ALT), aspartate aminotransferase (AST) and other liver biochemistry markers were assayed by Beckman’s wholly-automatic biochemical analyzer (Beckman Coulter, CA, USA). Two patients’ ALT data were excluded because of inconsistent time point of sampling.

### Quantitative detection of HBV DNA

HBV DNA was extracted from the serum of chronic hepatitis B patients. DNA level in the blood was detected by the real-time fluorescent quantitative polymerase chain reaction (PCR) (Da’An GENE, Guangzhou, China) on ABI7500 (Applied Biosystems, Foster City, USA). The detection sensitivity was as low as 500 copies/mL.

### Amplification and sequencing of HBV RT region

HBV DNA was extracted from the sera of patients using QIAamp DNA Blood Kit (Qiagen, Hilden, Germany). RT region was amplified by nested PCR as previously described[18]. The primers used in the first round PCR were 5-AGTCAGGAAGACAGCCTACTCC-3 (nt 3146–3167) and 5-AGGTGAAGCGAAGTGCACAC-3(nt 1577–1596); the primers used in the second round PCR were 5-TTCCTGCTGGTGGCTCCAGTTC-3 (nt 54–75) and 5-TTCCGCAGTATGGATCGGCAG-3 (nt 1258–1278). PCR products were purified using the QIAquick Gel Extraction Kit (Qiagen, Hilden, Germany) and sequenced commercially (Sangon Biotech, Shanghai, China). Nucleotide sequences were analyzed using DNAStar 5.0 and MEGA 4.0 softwares. Mutations in HBV RT region were determined by sequence alignment of PCR products with the reference stains in GenBank[6].

### Statistical analysis

Statistical analyses were performed using SPSS17.0 software (SPSS, Chicago, IL). Between-group comparisons were performed using one-way ANOVA *t* test or chi-square test, as appropriate. Serum HBV DNA concentrations were expressed on a logarithmic scale. A *P* value of less than 0.05 was considered statistically significant.

## References

[1] Ganem D, Prince AM. (2004) Hepatitis B virus infection—natural history and clinical consequences. N Engl J Med 350: 1118–1129. 15014185 10.1056/NEJMra031087

[2] Lu FM, Zhuang H. (2009) Management of hepatitis B in China. Chin Med J (Engl) 122: 3–4. 19187608

[3] Nassal M. (2008) Hepatitis B viruses: reverse transcription a different way. Virus Res 134: 235–249. 10.1016/j.virusres.2007.12.024 18339439

[4] Nowak MA, Bonhoeffer S, Hill AM, Boehme R, Thomas HC, et al (1996) Viral dynamics in hepatitis B virus infection. Proc Natl Acad Sci U S A 93: 4398–4402. 8633078 10.1073/pnas.93.9.4398 PMC39549

[5] Sheldon J, Soriano V. (2008) Hepatitis B virus escape mutants induced by antiviral therapy. J Antimicrob Chemother 61: 766–768. 10.1093/jac/dkn014 18218641

[6] Stuyver LJ, Locarnini SA, Lok A, Richman DD, Carman WF, et al (2001) Nomenclature for antiviral-resistant human hepatitis B virus mutations in the polymerase region. Hepatology 33: 751–757. 11230757 10.1053/jhep.2001.22166

[7] Sheldon J, Rodès B, Zoulim F, Bartholomeusz A, Soriano V. (2006) Mutations affecting the replication capacity of the hepatitis B virus. J Viral Hepat 13: 427–434. 16792535 10.1111/j.1365-2893.2005.00713.x

[8] Torresi J. (2002) The virological and clinical significance of mutations in the overlapping envelope and polymerase genes of hepatitis B virus. J Clin Virol 25: 97–106. 12367644 10.1016/s1386-6532(02)00049-5

[9] Zhang M, Ge G, Yang Y, Cai X, Fu Q, et al (2013) Decreased antigenicity profiles of immune-escaped and drug-resistant hepatitis B surface antigen (HBsAg) double mutants. Virol J 10: 292 10.1186/1743-422X-10-292 24053482 PMC3856468

[10] Selabe SG, Lukhwareni A, Song E, Leeuw YG, Burnett RJ, et al (2007) Mutations associated with lamivudine-resistance in therapy-naive hepatitis B virus (HBV) infected patients with and without HIV co-infection: implications for antiretroviral therapy in HBV and HIV co-infected South African patients. J Med Virol 79: 1650–1654. 17854040 10.1002/jmv.20974

[11] Rodriguez C, Chevaliez S, Bensadoun P, Pawlotsky JM. (2013) Characterization of the dynamics of hepatitis B virus resistance to adefovir by ultra-deep pyrosequencing. Hepatology 58: 890–901. 10.1002/hep.26383 23505208

[12] Tenney DJ, Rose RE, Baldick CJ, Pokornowski KA, Eggers BJ, et al (2009) Long-term monitoring shows hepatitis B virus resistance to entecavir in nucleoside-naïve patients is rare through 5 years of therapy. Hepatology 49: 1503–1514. 10.1002/hep.22841 19280622

[13] Zhang Y, Lian JQ, Li Y, Wang JP, Huang CX, et al (2013) Telbivudine plus adefovir therapy for chronic hepatitis B patients with virological breakthrough or genotypic resistance to telbivudine. Eur J Gastroenterol Hepatol 25: 814–819. 10.1097/MEG.0b013e32835ee516 23406845

[14] Leung N. (2002) Treatment of chronic hepatitis B: case selection and duration of therapy. J Gastroenterol Hepatol 17: 409–414. 11982721 10.1046/j.1440-1746.2002.02767.x

[15] Angus P, Vaughan R, Xiong S, Yang H, Delaney W, et al (2003) Resistance to adefovir dipivoxil therapy associated with the selection of a novel mutation in the HBV polymerase. Gastroenterology 125: 292–297. 12891527 10.1016/s0016-5085(03)00939-9

[16] Ji D1, Liu Y, Li L, Xu Z, Si LL, et al (2012) The rtL229 substitutions in the reverse transcriptase region of hepatitis B virus (HBV) polymerase are potentially associated with lamivudine resistance as a compensatory mutation. J Clin Virol 54: 66–72. 10.1016/j.jcv.2012.02.003 22398037

[17] Han KH, Hong SP, Choi SH, Shin SK, Cho SW, et al (2011) Comparison of multiplex restriction fragment mass polymorphism and sequencing analyses for detecting entecavir resistance in chronic hepatitis B. Antivir Ther 16: 77–87. 10.3851/IMP1702 21311111

[18] Liu Y, Wang C, Zhong Y, Li X, Dai J, et al (2011) Genotypic resistance profile of hepatitis B virus (HBV) in a large cohort of nucleos(t)ide analogue-experienced Chinese patients with chronic HBV infection. J Viral Hepat 18: e29–39. 10.1111/j.1365-2893.2010.01360.x 21392168 PMC7167191

[19] Liu BM, Li T, Xu J, Li XG, Dong JP, et al (2010) Characterization of potential antiviral resistance mutations in hepatitis B virus reverse transcriptase sequences in treatment-naïve Chinese patients. Antiviral Res 85(3): 512–519. 10.1016/j.antiviral.2009.12.006 20034521

[20] Ciftci S, Keskin F, Cakiris A, Akyuz F, Pinarbasi B, et al (2014) Analysis of potential antiviral resistance mutation profiles within the HBV reverse transcriptase in untreated chronic hepatitis B patients using an ultra-deep pyrosequencing method. Diagn Microbiol Infect Dis 79(1): 25–30. 10.1016/j.diagmicrobio.2014.01.005 24630522

[21] Sayan M, Akhan SC, Senturk O. (2011) Frequency and mutation patterns of resistance in patients with chronic hepatitis B infection treated with nucleos(t)ide analogs in add-on and switch strategies. Hepat Mon 11: 835–842. 10.5812/kowsar.1735143X.775 22224083 PMC3234585

[22] Zoulim F, Locarnini S. (2009) Hepatitis B virus resistance to nucleos(t)ide analogues. Gastroenterology 137: 1593–1608. 10.1053/j.gastro.2009.08.063 19737565

[23] Pollicino T, Isgrò G, Di Stefano R, Ferraro D, Maimone S, et al (2009) Variability of reverse transcriptase and overlapping S gene in hepatitis B virus isolates from untreated and lamivudine-resistant chronic hepatitis B patients. Antivir Ther 14:649–654. 19704167

[24] Singla B, Chakraborti A, Sharma BK, Kapil S, Chawla YK, et al (2013) Hepatitis B virus reverse transcriptase mutations in treatment Naïve chronic hepatitis B patients. J Med Virol 85(7):1155–1162. 10.1002/jmv.23608 23918533

[25] Huang ZM, Huang QW, Qin YQ, He YZ, Qin HJ, et al (2005) YMDD mutations in patients with chronic hepatitis B untreated with antiviral medicines. World J Gastroenterol 11(6): 867–870. 15682483 10.3748/wjg.v11.i6.867 PMC4250599

[26] Wang F, Wang H, Shen H, Meng C, Weng X, et al (2009) Evolution of hepatitis B virus polymerase mutations in a patient with HBeAg-positive chronic hepatitis B virus treated with sequential monotherapy and add-on nucleoside/nucleotide analogues. Clin Ther 31(2): 360–366. 10.1016/j.clinthera.2009.02.016 19302908

[27] Zöllner B, Sterneck M, Wursthorn K, Petersen J, Schröter M, et al (2005) Prevalence, incidence, and clinical relevance of the reverse transcriptase V207I mutation outside the YMDD motif of the hepatitis B virus polymerase during lamivudine therapy. J Clin Microbiol 43(5):2503–2505. 15872296 10.1128/JCM.43.5.2503-2505.2005 PMC1153772

[28] Ji D, Liu Y, Li L, Xu Z, Si LL, et al (2012) The rtL229 substitutions in the reverse transcriptase region of hepatitis B virus (HBV) polymerase are potentially associated with lamivudine resistance as a compensatory mutation. J Clin Virol 54(1):66–72. 10.1016/j.jcv.2012.02.003 22398037

[29] Liu Y, Li X, Xin S, Xu Z, Chen R, et al. (2014) The rtA181S mutation of hepatitis B virus primarily confers resistance to adefovir dipivoxil. J Viral Hepat doi: 10.1111 [Epub ahead of print] 10.1111/jvh.12298 25132017

[30] Ryu SH, Chung YH. (2006) Resistance to adefovir in patients with chronic hepatitis B. Korean J Hepatol 12(4): 484–492. 17237626

[31] Yang H, Westland CE, Delaney WE 4th, Heathcote EJ, Ho V, et al (2002) Resistance surveillance in chronic hepatitis B patients treated with adefovir dipivoxil for up to 60 weeks. Hepatology 36(2): 464–473. 12143057 10.1053/jhep.2002.34740

[32] Cento V, Van Hemert F, Neumann-Fraune M, Mirabelli C, Di Maio VC, et al (2013) Anti-HBV treatment induces novel reverse transcriptase mutations with reflective effect on HBV S antigen. J Infect 67(4):303–12. 10.1016/j.jinf.2013.05.008 23796863

[33] Torresi J. (2002) The virological and clinical significance of mutations in the overlapping envelope and polymerase genes of hepatitis B virus. J Clin Virol 25(2):97–106. 12367644 10.1016/s1386-6532(02)00049-5

[34] Liu Y, Li X, Xin S, Xu Z, Chen R, et al. (2014) The rtA181S mutation of hepatitis B virus primarily confers resistance to adefovir dipivoxil. J Viral Hepat 10.1111/jvh.12298 [Epub ahead of print] 25132017

[35] Fan J, Wang Y, Xiong H, Guo X, Cheng YC. (2014) Impact of the rtI187V polymerase substitution of hepatitis B virus on viral replication and antiviral drug susceptibility. J Gen Virol 95(Pt 11):2523–2530. 10.1099/vir.0.066886-0 25028473 PMC4202270

[36] Dandri M, Locarnini S. (2012) New insight in the pathobiology of hepatitis B virus infection. Gut 61 Suppl: i6–17. 10.1136/gutjnl-2012-302056 22504921

[37] Ou JH, Laub O, Rutter WJ. (1986) Hepatitis B virus gene function: the precore region targets the core antigen to cellular membranes and causes the secretion of the e antigen. Proc Natl Acad Sci U S A 83: 1578–1582. 3006057 10.1073/pnas.83.6.1578 PMC323126

[38] Wang J, Lee AS, Ou JH. (1991) Proteolytic conversion of hepatitis B virus e antigen precursor to end product occurs in a postendoplasmic reticulum compartment. J Virol 65: 5080–5083. 1870212 10.1128/jvi.65.9.5080-5083.1991 PMC248973

[39] Ciupe SM, Hews S. (2012) Mathematical models of e-antigen mediated immune tolerance and activation following prenatal HBV infection. PLoS One 7: e39591 10.1371/journal.pone.0039591 22768303 PMC3388102

[40] Milich DR, Schödel F, Hughes JL, Jones JE, Peterson DL. (1997) The hepatitis B virus core and e antigens elicit different Th cell subsets: antigen structure can affect Th cell phenotype. J Virol 71: 2192–2201. 9032353 10.1128/jvi.71.3.2192-2201.1997 PMC191326

[41] Milich DR, Chen MK, Hughes JL, Jones JE. (1998) The secreted hepatitis B precore antigen can modulate the immune response to the nucleocapsid: a mechanism for persistence. J Immunol 160: 2013–2021. 9469465

[42] Wang YX, Xu X, Luo C, Ma ZM, Jiang HL, et al (2007) A putative new domain target for anti-hepatitis B virus: residues flanking hepatitis B virus reverse transcriptase residue 306 (rtP306). J Med Virol 79: 676–682. 17457904 10.1002/jmv.20835

[43] Melegari M, Scaglioni PP, Wands JR. (1998) Hepatitis B virus mutants associated with 3TC and famciclovir administration are replication defective. Hepatology 27: 628–633. 9462667 10.1002/hep.510270243

[44] Amini-Bavil-Olyaee S, Herbers U, Sheldon J, Luedde T, Trautwein C, et al (2009) The rtA194T polymerase mutation impacts viral replication and susceptibility to tenofovir in hepatitis B e antigen-positive and hepatitis B e antigen-negative hepatitis B virus strains. Hepatology 49: 1158–1165. 10.1002/hep.22790 19263474

[45] Zhu Y, Curtis M, Borroto-Esoda K. (2011) The YMDD and rtA194T mutations result in decreased replication capacity in wild-type HBV as well as in HBV with precore and basal core promoter mutations. Antivir Chem Chemother 22: 13–22. 10.3851/IMP1791 21860069

